# Severity of nausea and vomiting in pregnancy and early childhood neurobehavioural outcomes: The Growing Up in Singapore Towards Healthy Outcomes study

**DOI:** 10.1111/ppe.12703

**Published:** 2020-06-23

**Authors:** Nicholas L. Syn, Shiao‐Yng Chan, Elisha Wan Ying Chia, Wei Xin Ong, Desiree Phua, Shirong Cai, Lynette P. C. Shek, Yap‐Seng Chong, Lourdes Mary Daniel, Birit F. P. Broekman, Keith M. Godfrey, Michael J. Meaney, Evelyn C. Law

**Affiliations:** ^1^ Yong Loo Lin School of Medicine National University of Singapore Singapore Singapore; ^2^ Department of Obstetrics and Gynaecology National University Health System Singapore Singapore; ^3^ Agency for Science, Technology and Research (A*STAR) Singapore Institute for Clinical Sciences (SICS) Singapore Singapore; ^4^ Department of Paediatrics Yong Loo Lin School of Medicine National University of Singapore Singapore Singapore; ^5^ Khoo Teck Puat‐National University Children's Medical Institute National University Health System Singapore Singapore; ^6^ Department of Child Development KK Women’s and Children’s Hospital Singapore Singapore; ^7^ Duke‐NUS Graduate Medical School Singapore Singapore; ^8^ Department of Psychiatry VU University Medical Centre Amsterdam Netherlands; ^9^ MRC Lifecourse Epidemiology Unit & NIHR Southampton Biomedical Research Centre University of Southampton and University Hospital Southampton NHS Foundation Trust UK; ^10^ Ludmer Centre for Neuroinformatics and Mental Health Douglas Institute McGill University Montreal Canada; ^11^ Departments of Psychiatry and Neurology and Neurosurgery McGill University Montreal Canada; ^12^ Sackler Program for Epigenetics and Psychobiology McGill University Montreal Canada

**Keywords:** behaviours, child development, hyperemesis gravidarum, Nausea in pregnancy, vomiting in pregnancy

## Abstract

**Background:**

Nausea and vomiting of pregnancy (NVP) affects 50 to 80 per cent of women. The existing literature has examined NVP from the perspective of the mother, and relatively less is known about offspring outcomes.

**Objectives:**

To study the relationships of NVP with social‐emotional, behavioural, and cognitive outcomes of the offspring in a multi‐ethnic Asian cohort.

**Methods:**

In the Growing Up in Singapore Towards Healthy Outcomes prospective mother‐offspring cohort study, mothers responded to a structured NVP questionnaire at 26‐28 weeks’ gestation (n = 1172) and participants with severe NVP were confirmed using medical records. Children underwent multiple neurodevelopmental assessments throughout childhood. We conducted multivariable regressions with post‐estimation predictive margins to understand the associations of NVP with offspring neurobehavioural outcomes, which included 1‐year Infant‐Toddler Social and Emotional Assessment, 1.5‐year Quantitative Checklist for Autism in Toddlers, 2‐year Bayley Scales of Infant and Toddler Development, 2‐ and 4‐year Child Behavior Checklist, and 4.5‐year Kaufman Brief Intelligence Test. Analyses were adjusted for household income, birth variables, maternal mental health, and other relevant medical variables. Cohen's *d* effect sizes were calculated using standardised mean differences (μ_d_).

**Results:**

Mothers were categorised into no (n = 296, 25.3%), mild‐moderate (n = 686, 58.5%), and severe NVP (n = 190, 16.2%), of whom 67 (5.7%) required admission. Compared to children of mothers who had no or mild‐moderate NVP, children with exposure to severe NVP exhibited more externalising behaviours (μ_d_ 2.0, 95% CI 0.3, 3.6; Cohen's *d* = 0.33) and social communication difficulties before 2 years (μ_d_ 4.1, 95% Cl 0.1, 8.0; Cohen's *d* = 0.38), both externalising (μ_d_ 1.5, 95% CI 0.4, 2.6; Cohen's *d* = 0.43) and internalising behaviours at 2 years (μ_d_ 1.2, 95% CI 0.1, 2.2; Cohen's *d* = 0.35), and only internalising behaviours after 2 years (μ_d_ 1.1, 95% CI 0.4, 2.0; Cohen's *d* = 0.37).

**Conclusions:**

Severe NVP is highly prevalent in this Asian cohort and may be adversely associated with multiple offspring neurobehavioural outcomes.

**Synopsis**

1 **Study question**

What is the relationship of nausea and vomiting in pregnancy (NVP) with the social‐emotional, behavioural, and cognitive development of the offspring?

2 **What's already known**

The existing literature demonstrates the psychosocial burden of NVP on pregnant mothers. A few studies have shown the contribution of NVP to emotional behavioural difficulties in school‐age and adult offspring. Neurobehavioural outcomes in early childhood have not been thoroughly explored.

3 **What this study adds**

Severe NVP in this multi‐ethnic Asian cohort correlates with a variety of neurobehavioural impairments in the offspring. The behavioural phenotype shifts from predominantly externalising symptoms in the first 2 years to internalising symptoms after 2 years of life. No associations are found between NVP and cognitive intelligence at 4.5 years.

## 1 INTRODUCTION

Nausea and vomiting of pregnancy (NVP) is characterised by a spectrum of severity and affects 50 to 80 per cent of women during pregnancy.^1^, ^2^, ^3^ Symptoms typically peak during the first trimester and remit by the 20th week of gestation.^4^, ^5^, ^6^ The pathophysiology of NVP remains incompletely understood but is likely multifactorial with hormonal, environmental, psychological, evolutionary, and genetic etiologies.^4^, ^5^, ^6^ Hyperemesis gravidarum represents the most morbid clinical presentation across the spectrum of NVP and has been variably defined as persistent nausea and vomiting leading to weight loss in excess of 5% of pre‐pregnancy weight^2^, ^7^ and/or warranting hospital admission based on the Fairweather criteria. The Fairweather criteria define hyperemesis gravidarum as “vomiting occurring in pregnancy before the 20th week of gestation, not associated with coincidental conditions as appendicitis, pyelitis, etc, and of such severity as to require the patient's admission to hospital.”^8^ The reported incidence of hyperemesis gravidarum is 0.3 to 1.0 per cent in Caucasian populations^9^, ^10^ and up to 3.0 to 10.8 per cent in Asian populations,^4^, ^6^ hinting at potentially distinct biological mechanisms governing NVP in different races, ethnic aggregation, or genetic susceptibility.^11^

Hyperemesis gravidarum and its impact on maternal physical and mental well‐being have been extensively studied. Hitherto, much of the existing literature on the psychosocial burden of NVP has been examined from the perspective of the mother,^12^, ^13^, ^14^, ^15^, ^16^ and relatively less is known of pregnancy and offspring outcomes. Whereas milder symptoms have been considered inconsequential or even protective of adverse outcomes such as miscarriage,^17^, ^18^, ^19^, ^20^ accumulating evidence has suggested a link between hyperemesis gravidarum and placental dysfunction disorders, including preeclampsia, abruption, stillbirth, small for gestational age, and preterm birth.^13^, ^21^ Considering that weight loss, poor diet quality, nutritional deficiencies, and ketonuria are complications after protracted vomiting, hyperemesis gravidarum imposes physiological and metabolic stresses which recapitulate a state of starvation or malnutrition in utero, which in turn has been postulated to account for the developmental origins of many adult diseases.^22^

Prenatal exposure to hyperemesis gravidarum has been reportedly associated with mental health disorders in adult offspring, as well as a variety of attention, learning, and language problems in children.^1^, ^20^, ^23^, ^24^, ^25^, ^26^, ^27^ Two studies have reported differences in developmental outcomes only for protracted NVP that continued into the mid‐late second trimester.^1^, ^24^ Other studies have also emerged recently that described the relationship between NVP and autism spectrum disorders.^28^, ^29^, ^30^, ^31^ Accordingly, this study was conducted in a prospective mother‐offspring cohort to examine a wider range of early childhood outcomes, consisting of social‐emotional, behavioural, and cognitive outcomes, after exposure to different severity of NVP in utero.

## 2 METHODS

### 2.1 Study population

The Growing Up in Singapore Towards Healthy Outcomes (GUSTO) cohort is a population‐based, prospective longitudinal study of pregnancies and children with the aim of studying how antenatal and early childhood conditions influence the long‐term health of women and children. Briefly, women aged 18 years or older across all socioeconomic backgrounds were recruited from KK Women's and Children's Hospital (KKH) and National University Hospital (NUH) in Singapore between 2009 and 2010 during their first trimester of pregnancy, as described previously.^32^ GUSTO inclusion criteria required both biological parents to be one of the three major ethnicities in Singapore (ie Chinese, Malay or Indian) and are Singaporeans (ie not recent immigrants). Participants were followed up through and beyond delivery, and their children tested regularly over childhood.

### 2.2 Exposures

Severity of vomiting in the first and second trimester was recorded in a structured interview‐administered questionnaire at 26‐28 weeks’ gestation. Electronic medical records maintained by the hospitals were utilised to confirm the severity of vomiting, including prescription of anti‐emetic medications and hospital admissions. Seventy‐four women were admitted due to non‐infectious nausea and vomiting and all of them had “hyperemesis gravidarum” as the discharge diagnosis. Congruence between mothers’ reports and medical records for severe NVP was 90.5%. Sixty‐seven participants with congruent records were included. As weight loss was not systematically obtained, we did not categorise pregnant mothers into hyperemesis gravidarum. Instead, the severity of nausea and vomiting was classified into none, mild‐moderate (defined as nausea and/or vomiting occasionally), and severe (defined as regular vomiting with inability to retain meals).

### 2.3 Outcomes

The outcomes analysed in this study were scores from the 1‐year Infant‐Toddler Social and Emotional Assessment (ITSEA; n = 542)^33^; 1.5‐year Quantitative Checklist for Autism in Toddler (Q‐CHAT; n = 208),^34^, ^35^ 2‐year Bayley Scales of Infant and Toddler Development, Third Edition (Bayley‐III; n = 397),^36^ 2‐ and 4‐year Child Behavior Checklist (CBCL; 2‐year n = 506, 4‐year mother n = 666, 4‐year father n = 586),^37^ and the Kaufman Brief Intelligence Test, Second Edition (KBIT‐2) at 4.5 years (n = 476).^38^ The cognitive assessments were completed by blinded research staff, and the questionnaires were completed by mothers, except for the 4‐year CBCL, which were completed by both mothers and fathers individually. The inclusion of father‐reported CBCL was to reduce any reporter bias that might arise when mothers report both the exposure and the outcome. To avoid dependency and intra‐subject correlation in the neurobehavioural outcomes of multiple‐birth siblings and repeated measurement of maternal variables, we only included mothers with singleton pregnancies.

### 2.4 Statistical analyses

Multivariable linear regression models were used to delineate the relationships between NVP and child outcomes, and analyses were adjusted for the following covariates: gestational age, birthweight (World Health Organization (WHO) gestational age and sex‐specific Z‐scores), maternal age at delivery, monthly household income, gestational diabetes (defined by WHO 1999 criteria), hypertensive disorders of pregnancy (ie preeclampsia, eclampsia, or pregnancy‐induced hypertension), positive smoking or tobacco smoke exposure (self‐reported or plasma cotinine blood test > 0.17 ng/mL), sex of baby, and the changes in maternal general mood from pregnancy to 3‐months post‐partum. The maternal general mood variable was taken from an exploratory bifactor analysis (EFA) combining items from the State‐Trait Anxiety Inventory (STAI),^39^, ^40^ Beck Depression Inventory (BDI‐II),^41^, ^42^ and Edinburgh Postnatal Depression Scale (EPDS),^43^, ^44^ as reported previously.^45^ Briefly, this EFA was fitted with the individual items of the mental health scales as manifest variables, and factors were retained if the eigenvalue from observed data was larger than eigenvalues from parallel analysis (1000 randomly generated correlation matrices).^45^ An oblique rotation (bi‐geomin) was used to permit correlation between subfactors, and estimates were obtained under maximum likelihood with robust standard errors.^45^

Unadjusted mean scores and their 95% confidence intervals for the three categories of NVP were obtained using the post‐estimation predictive margins command immediately after fitting regression models. To further facilitate interpretation regarding the clinical significance, we reported Cohen's *d* for effect sizes. Statistical analyses were completed in Stata version 16.0 (StataCorp).^46^

### 2.5 Sensitivity analyses

Since women with severe NVP were grouped together in the multivariable regression regardless of admission into the hospital, we conducted sensitivity analyses to test the uncertainty around whether there were differences in child outcomes between mothers with severe NVP who sought care in the hospital versus those who did not. We found that the 123 mothers with severe NVP who were not hospitalised accounted for our results in Tables 2 and 3, and not the 67 women with severe NVP who were hospitalised (Table S1).

### 2.6 Missing data

Our missing data tabulation showed that 37% of children born to mothers with NVP data did not have data related to neurodevelopment, while only 15% had dropped out entirely from the GUSTO cohort at 4.5 years. This was because approximately 22% of the children in the cohort were not asked to attend the neurodevelopment visits, as a way to reduce time burden on the families. Those who attended the neurodevelopmental visits were randomly selected from the 1172 mothers, except for the 4‐year visit, when children with more consistent attendance in prior visits were prioritised to ensure a higher sample of complete data. To assess for potential bias due to missing data, we compared the demographic characteristics and the NVP distribution of mothers whose children had missing or non‐missing outcomes. We did not find any differences between the two groups, except for a small difference in ethnicity at the 1‐year follow‐up visit (Table S2).

Missing data were handled by multiple imputations (*m* = 50) on the independent and dependent variables using chained equations. Before re‐calculating the multivariable regression estimates from the imputed data, we deleted imputed data of the dependent variables and left only the observed data to minimise bias introduced by a misspecified imputation model for the outcomes.^47^

### 2.7 Ethics approval

The study protocol was approved by the SingHealth Centralized Institutional Review Board and National Healthcare Group Domain Specific Review Board, and all participants gave written informed consent prior to study enrolment.

## 3 RESULTS

### 3.1 Description of cohort

**FIGURE 1:**
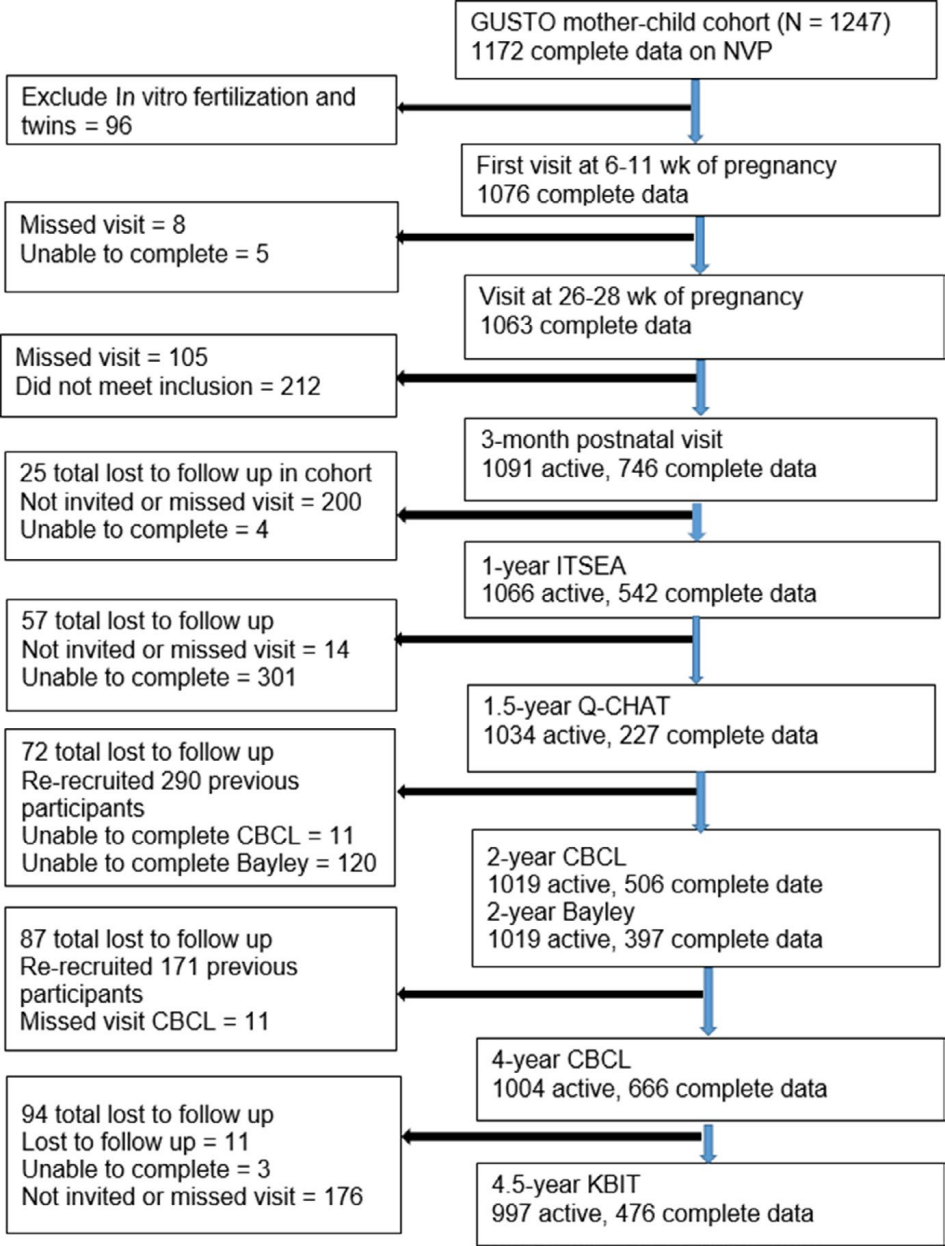
Study flow diagram of the population‐based cohort

Of 1247 mother‐child dyads in the GUSTO study, 1172 mothers completed a structured interview‐administered questionnaire at 26‐28 weeks’ gestation with information about first and second trimester nausea and vomiting (Figure 1). Altogether 296 (25.3%) women reported no vomiting, 686 (58.5%) had mild or moderate vomiting, and 190 (16.2%) reported severe vomiting. Of the 74 women classified as severe vomiting and required rehydration in the hospital for pregnancy‐related vomiting, 67 (90.5%) of them reported congruent degree of NVP as the hospital records. Baseline maternal demographics, adverse intra‐uterine exposures (including smoking exposure, hypertensive disorders of pregnancy, and gestational diabetes mellitus), pre‐ and postnatal mood scores of mothers, and birthweight were comparable across women with varying degrees of NVP (Table 1).

**TABLE 1:** Characteristics of participants in the Growing Up Towards Healthy Outcomes (GUSTO) study and relative risks between mothers with severe vomiting and no vomiting

Characteristic	Severity of vomiting			Relative risk (95% confidence interval)
	None (n = 296)	Mild‐Moderate (n = 686)	Severe (n = 190)	
Maternal age, median (IQR), years	31.3 (27.9‐35.3)	31.2 (27.6‐34.9)	31.3 (27.2‐34.6)	—
Gestational age, median (IQR), week	39.0 (38.1‐39.7)	38.9 (38.0‐39.7)	38.7 (37.6‐39.6)	—
Pregnancy Edinburgh postnatal depression scale (EPDS) score, mean (SD)	6.93 (4.54)	7.55 (4.30)	7.86 (4.91)	—
Pregnancy state‐trait anxiety inventory (STAI) score, mean (SD)	70.04 (18.29)	71.42 (17.65)	72.54 (18.04)	—
3‐month postnatal EPDS score, mean (SD)	5.86 (4.43)	6.54 (4.69)	7.13 (5.23)	—
3‐month postnatal STAI score, mean (SD)	69.0 (18.4)	70.8 (19.4)	71.5 (20.4)	—
Birthweight in kilograms, mean (SD)	3.08 (0.43)	3.11 (0.56)	3.03 (0.46)	—
Birthweight z‐score, mean (SD)^a^	0.06 (1.17)	0.179 (1.25)	0.155 (1.21)	—
Maternal race, %				
Chinese	58.5	58.2	47.3	1.00 (Reference)
Malay	22.3	26.5	26.1	1.44 (0.92, 2.26)
Indian	19.3	15.3	26.6	1.71 (1.08, 2.70)
Smoke exposure in pregnancy, %	14.2	16.0	14.7	0.95 (0.66, 1.38)
Breast feeding for ≥3 mo, %	57.6	57.7	43.5	0.62 (0.47, 0.82)
Gestational diabetes mellitus, %	17.0	19.0	19.0	1.03 (0.73, 1.45)
Hypertensive disorders of pregnancy, %	7.8	5.4	6.3	1.03 (0.60, 1.76)
Household income per month (SGD $), %				
0‐1999	14.0	15.3	16.2	1.00 (Reference)
2000‐3999	30.9	28.5	38.0	1.06 (0.60, 1.89)
4000‐5999	25.9	25.3	22.4	0.75 (0.40, 1.38)
>6000	29.1	31.0	23.5	0.70 (0.38, 1.28)
Male child, %	54.0	55.2	41.9	0.65 (0.50, 0.84)

Note

Abbreviations: SGD$, Singapore Dollars.

^a^ Standardised for gestational age and sex.

However, there were differences in child's sex, ethnicity, gestational age, and breast feeding across the three categories of vomiting severity (Table 1). Mothers who conceived sons were, as a group, less likely to report severe NVP than mothers who conceived daughters. In our multi‐ethnic cohort, Indian mothers were more likely to report severe vomiting as compared with Chinese mothers, while Malay ethnicity did not appear to be associated with severity of vomiting. No inter‐group differences were found in maternal mood from antenatal to postnatal period based on ethnicity of the women. Finally, mothers with severe NVP were less likely to report breast feeding for at least 3 months as compared with mothers with no, mild, and moderate NVP, which was likely related to unmeasured confounders.

### 3.2 Child outcomes after exposure to nausea and vomiting in pregnancy

In our multivariable regression models, we observed more pronounced externalising behaviours at 1 year and more social communication difficulties at 1.5‐year in children born to mothers who reported severe vomiting as compared to those who reported no or milder NVP (Table 2). Dysregulated behaviours were also elevated at 1‐year, along with negative emotionality (ie tendency to react to stressful situations with unpleasant emotions) and sleep disturbances in infants born to mothers with severe NVP (Table 2). The models for data deleted listwise and for imputed data produced the same results.

**TABLE 2:** Severe NVP and its relationships with 1‐year ITSEA subscales and 1.5‐y Q‐CHAT total score

Outcome^a^	Severity of vomiting	Unadjusted means (95% CI)	Adjusted difference in means (95% CI)^b^	Cohen's *d*
Activity and impulsivity	None (n = 132)	5.2 (4.7, 5.7)	0.0 (Reference)	0.38
	Mild‐moderate (n = 312)	5.2 (4.9, 5.5)	0.0 (−0.6, 0.6)	
	Severe (n = 81)	6.0 (5.4, 6.7)	0.9 (0.1, 1.7)	
Externalising Behaviours	None (n = 126)	11.1 (9.8, 12.3)	0.0 (Reference)	0.33
	Mild‐moderate (n = 307)	10.6 (9.8, 11.3)	−0.5 (−2.0, 1.0)	
	Severe (n = 81)	12.6 (11.1, 14.0)	2.0 (0.3, 3.6)	
Negative emotionality	None (n = 131)	6.7 (5.7, 7.7)	0.0 (Reference)	0.37
	Mild‐moderate (n = 317)	7.1 (6.5, 7.7)	0.4 (−0.8, 1.5)	
	Severe (n = 85)	8.9 (7.3, 9.6)	1.8 (0.2, 3.3)	
Sleep problems	None (n = 130)	2.4 (1.9, 2.8)	0.0 (Reference)	0.47
	Mild‐moderate (n = 315)	2.8 (2.6, 3.1)	0.5 (−0.1, 1.0)	
	Severe (n = 85)	3.3 (2.7, 3.8)	0.9 (0.2, 1.6)	
Dysregulation domain	None (n = 131)	17.0 (15.1, 18.8)	0.0 (Reference)	0.41
	Mild‐moderate (n = 316)	17.3 (16.2, 18.4)	0.3 (−1.9, 2.5)	
	Severe (n = 85)	20.0 (17.8, 22.3)	3.1 (0.1, 6.0)	
Q‐CHAT total score	None (n = 54)	34.7 (32.4, 37.0)	0.0 (Reference)	0.38
	Mild‐moderate (n = 150)	33.5 (32.2, 37.9)	−1.2 (−3.9, 1.5)	
	Severe (n = 24)	38.8 (35.5, 42.0)	4.1 (0.1, 8.0)	

^a^ The ITSEA questionnaire comprises numerous subscales; only domains with meaningful effect sizes based on inspection of 95% confidence intervals are displayed in this table.

^b^ Covariates included gestational age, birthweight Z‐scores, maternal age, household monthly income, gestational diabetes, hypertensive disorders of pregnancy, history of smoking exposure and/or positive plasma cotinine, sex of child, maternal general mood factor from pregnancy to 3‐mo post‐partum, and parity.

It is important to note that regression models were consecutively run for all domains and subscales (over 30 in total) in the ITSEA and CBCL without selective searching for notable associations. We elected to display only domains with meaningful effect sizes based on inspection of 95% confidence intervals. The reader may therefore interpret all ITSEA and CBCL outcomes not shown in the tables as having 95% confidence intervals that crossed zero.

To afford a more comprehensive and longitudinal assessment, we used the Childhood Behavior Checklist (CBCL) at 2‐ and 4‐year time points (Table 3). At age 2, we found more externalising and internalising problems in children with exposure to severe NVP as compared to those whose mothers reported no or mild‐moderate NVP during pregnancy. The most prominent areas of difficulty were related to regulation of emotions and attention, for example emotional reactivity and attention problems. In addition, consistent with age 1, higher scores in sleep problems were reported. In the DSM‐oriented scales, children exposed to severe NVP had higher symptoms of attention‐deficit hyperactivity disorder and affective disorder.

**TABLE 3:** Severe NVP and its relationship with 2‐y mother‐rated behaviours, 4‐y father‐reported behaviours, and 4‐year mother‐reported behaviours from CBCL

Outcome^a^	Severity of vomiting	Unadjusted means (95% CI)	Adjusted difference in means (95% CI)^b^		Cohen's *d*
			Observed	Imputed	
2‐y emotionally reactive	None (n = 93)	1.6 (1.0, 2.1)	0.0 (Reference)		0.43
	Mild‐moderate (n = 245)	2.1 (1.8, 2.4)	0.5 (−0.1, 1.2)	0.5 (−0.1, 1.2)	
	Severe (n = 55)	2.7 (2.0, 3.4)	1.1 (0.3, 2.0)	1.1 (0.2, 2.0)	
2‐y attention problems	None (n = 93)	2.5 (2.0, 3.0)	0.0 (Reference)		0.49
	Mild‐moderate (n = 245)	2.9 (2.7, 3.2)	0.4 (−0.1, 1.0)	0.4 (−0.1, 1.0)	
	Severe (n = 55)	3.7 (3.1, 4.3)	1.2 (0.4, 2.0)	1.2 (0.4, 2.0)	
2‐y sleep problems	None (n = 93)	2.6 (2.0, 3.3)	0.0 (Reference)		0.35
	Mild‐moderate (n = 245)	2.9 (2.6, 3.3)	0.3 (−0.4, 1.0)	0.3 (−0.4, 1.0)	
	Severe (n = 55)	3.7 (2.9, 4.5)	1.1 (0.1, 2.1)	1.1 (0.1, 2.1)	
2‐y DSM attention‐deficit	None (n = 93)	4.3 (3.6, 5.0)	0.0 (Reference)		0.43
	Mild‐moderate (n = 245)	4.9 (4.5, 5.3)	0.6 (−0.2, 1.4)	0.6 (−0.2, 1.4)	
	Severe (n = 55)	5.8 (4.9, 6.7)	1.5 (0.4, 2.6)	1.5 (0.4, 2.6)	
2‐y DSM affective	None (n = 93)	2.1 (1.4, 2.7)	0.0 (Reference)		0.35
	Mild‐moderate (n = 245)	2.4 (2.0, 2.8)	0.3 (−0.4, 1.1)	0.3 (−0.4, 1.1)	
	Severe (n = 55)	3.2 (2.4, 4.1)	1.2 (0.1, 2.2)	1.2 (0.1, 2.2)	
4‐y anxious‐depressive symptoms (paternal report)	None (n = 136)	2.7 (2.2, 3.2)	0.0 (Reference)		0.37
	Mild‐moderate (n = 354)	3.0 (2.6, 3.3)	0.2 (−0.4, 0.8)	0.2 (−0.4, 0.8)	
	Severe (n = 91)	3.8 (3.2, 4.5)	1.1 (0.3, 1.9)	1.1 (0.3, 1.9)	
4‐y anxious‐depressive symptoms (maternal report)	None (n = 152)	2.9 (2.4, 3.3)	0.0 (Reference)		0.28
	Mild‐moderate (n = 394)	2.8 (2.5, 3.1)	−0.1 (−0.6, 0.5)	−0.1 (−0.6, 0.5)	
	Severe (n = 111)	3.7 (3.1, 4.3)	0.6 (0.0, 1.2)	0.6 (0.0, 1.2)	
4‐y DSM affective (maternal report)	None (n = 152)	2.1 (1.6, 2.6)	0.0 (Reference)		0.22
	Mild‐moderate (n = 394)	2.4 (2.1, 2.7)	0.3 (−0.3, 0.9)	0.3 (−0.2, 0.9)	
	Severe (n = 111)	2.8 (2.3, 3.4)	0.8 (0.0, 1.5)	0.8 (0.0, 1.5)	
4‐y DSM anxiety (maternal report)	None (n = 152)	3.5 (3.0, 4.0)	0.0 (Reference)		0.37
	Mild‐moderate (n = 394)	3.7 (3.4, 4.0)	0.3 (−0.3, 0.9)	0.3 (−0.3, 0.9)	
	Severe (n = 111)	4.6 (4.0, 5.2)	1.1 (0.4, 2.0)	1.1 (0.3, 1.9)	

Abbreviations: DSM, Diagnostic and Statistical Manual for Mental Disorders.

^a^ The CBCL questionnaire comprises numerous subscales; only domains with meaningful effect sizes based on inspection of 95% confidence intervals are displayed in this table.

^b^ Covariates included gestational age, birthweight Z‐scores, maternal age, household monthly income, gestational diabetes, hypertensive disorders of pregnancy, history of smoking exposure and/or positive plasma cotinine, sex of child, maternal general mood factor from pregnancy to 3‐month post‐partum, and parity.

At 4 years, children exposed to severe NVP exhibited mainly internalising behaviours such as anxiety and depressive symptoms. It is notable that both mother and father corroborated this finding in separate reports. In addition, when compared to children born to mothers without or with milder NVP, those born to mothers who experienced severe vomiting during their pregnancy had more impairments based on several DSM‐oriented scales, including in affective and anxiety conditions (Table 3). We compared the models obtained from our imputed data with the observed data and found minimal changes in the estimates.

Severe vomiting was not associated with language and motor scores in 2‐year‐old children based on the Bayley‐III scale (Table 4). However, reductions of 5.4‐ and 5.1‐points in the non‐missing and imputed Bayley cognitive scores, respectively, were found, which corresponded to 1/3 of a standard deviation in this instrument. By 4.5 years of age, there were no differences in verbal, non‐verbal, and composite IQ scores based on the KBIT‐2 between the 3 groups of children. It is important to note that KBIT‐2 was conducted in English in this cohort. Since only 36.9% of Singaporean children speak English as their first language, the verbal IQ score (Mean 86.2, SD 16.1) was therefore much lower than non‐verbal IQ score (100.0, SD 14.7) at age 4.5.^48^

**TABLE 4:** A reduction in the Bayley cognitive score was found at 2 y in children with exposure to severe NVP. The cognitive scores in the KBIT‐2 at 4.5 y were not different based on severity of NVP

Outcome	Severity of vomiting	Unadjusted means (95% CI)	Adjusted difference in means (95% CI)^a^	
			Observed	Imputed
Bayley Composite cognitive (2 y)	None (n = 117)	105.6 (102.6, 108.5)	0.0 (Reference)	
	Mild‐moderate (n = 296)	103.0 (101.2, 104.7)	−2.6 (−6.0, 0.9)	−2.4 (−5.9, 1.2)
	Severe (n = 69)	100.2 (96.5, 103.9)	−5.4 (−10.1, −0.6)	−5.1 (−9.9, −0.3)
Bayley Composite language (2 y)	None (n = 117)	97.2 (93.8, 100.5)	0.0 (Reference)	
	Mild‐moderate (n = 296)	96.4 (94.5, 98.4)	−0.7 (−4.6, 3.2)	−0.7 (−4.6, 3.3)
	Severe (n = 69)	95.7 (91.6, 99.9)	−1.4 (−6.7, 3.9)	−1.4 (−6.7, 4.0)
Bayley Composite motor (2 y)	None (n = 115)	108.0 (104.8, 111.2)	0.0 (Reference)	
	Mild‐moderate (n = 291)	107.8 (106.0, 109.7)	−0.1 (−3.9, 3.6)	0.0 (−3.7, 3.8)
	Severe (n = 69)	103.2 (99.3, 107.1)	−4.8 (−9.8, 0.3)	−4.6 (−9.7, 0.4)
KBIT verbal (4.5 y)	None (n = 114)	87.0 (83.5, 90.6)	0.0 (Reference)	
	Mild‐moderate (n = 290)	86.0 (83.8, 88.1)	−1.1 (−5.2, 3.1)	−1.1 (−5.3, 3.1)
	Severe (n = 65)	86.2 (81.6, 90.8)	−0.9 (−6.6, 4.9)	−0.8 (−6.6, 5.0)
KBIT non‐verbal (4.5 y)	None (n = 114)	101.3 (98.0, 104.6)	0.0 (Reference)	
	Mild‐moderate (n = 293)	100.9 (98.9, 102.9)	0.3 (−4.2, 3.6)	0.2 (−3.7, 4.2)
	Severe (n = 65)	100.4 (96.1, 104.8)	−0.8 (−6.3, 4.6)	−0.3 (−5.8, 5.1)
KBIT IQ composite (4.5 y)	None (n = 114)	93.5 (90.3, 96.8)	0.0 (Reference)	
	Mild‐moderate (n = 290)	92.7 (90.7, 94.7)	−0.8 (−4.6, 3.1)	−0.4 (−4.3, 3.5)
	Severe (n = 65)	92.5 (88.2, 96.7)	−1.0 (−6.4, 4.4)	−0.7 (−6.1, 4.7)

^a^ Covariates included gestational age, birthweight Z‐scores, maternal age, household monthly income, gestational diabetes, hypertensive disorders of pregnancy, history of smoking exposure and/or positive plasma cotinine, sex of child, maternal general mood factor from pregnancy to 3‐month post‐partum, and parity.

## 4 COMMENT

### 4.1 Principal findings

In this large Asian prospective mother‐offspring cohort, children born to mothers with severe NVP during pregnancy showed worse emotional and behavioural functioning, as compared to children without exposure to NVP.

### 4.2 Strengths of the study

To date, there has been no other study using a non‐clinical cohort with such a wide range of outcomes in early childhood and in an Asian population, which is important considering potential biological and aetiological differences between women from different ethnic backgrounds. Our present study has important policy, clinical, and research implications, as our findings suggest that the in utero environment of fetuses exposed to severe NVP may set a trajectory of increased risk for poorer developmental outcomes. Other strengths include the prospective longitudinal study design, rigorous phenotyping of mother‐child dyads, adjustments for multiple covariates, and the use of validated tests for assessing early childhood neurocognitive and behavioural profiles.

### 4.3 Limitations of the data

Limitations of the study include self‐reporting of vomiting severity, which is subjective and may be associated with recall bias. However, medical record details have been used to ensure accuracy of the women's self‐report with a congruence of 90.5% in this study. Inherent in cohorts, there are up to 50 per cent of missing neurodevelopment data in the later years, which may bias our findings based on participants who are left in the cohort. Hence, we have employed multiple imputations and conducted sensitivity analyses. We did not find differences between our sample and the overall GUSTO cohort in terms of NVP categories of mothers, medical conditions during pregnancy, and child variables including gestational age and birthweight, except for a larger percentage of Singaporean Chinese families attending the 1‐year infant visit.

### 4.4 Interpretation

A novel finding from this study is that children born to mothers with severe NVP initially demonstrate mainly externalising symptoms in infancy and toddlerhood, while children present with more internalising symptoms by preschool age. Although the association between NVP and internalising symptoms in adult offspring has been consistently reported,^2^, ^9^, ^25^ little is known about internalising symptom profile from 1 to 4 years of age. In this study, the association of NVP and internalising symptoms can be observed as early as 2 to 4 years of age. Consistent with recent studies linking NVP and autism spectrum disorder, we have replicated similar results at 1.5‐years. Instead of using an ASD diagnosis as the outcome, this study uses social communication skills from the Q‐CHAT, which allows for a more granular understanding of the relationship between NVP and social communication as a continuum.

Severe NVP is highly prevalent in our cohort (16.2%), which has been suggested to be epidemiologically associated with Asian ethnicity.^4^, ^6^ Nevertheless, it is notable that rates have varied widely across studies. Possible explanations for between‐study heterogeneity in prevalence estimates include differences in the criteria used to define hyperemesis gravidarum, insufficient sample sizes, self‐reporting and recall bias, differences in the specific ethnic composition, and country‐specific sociocultural attitudes, guidelines and accessibility to health care institutions. It is intriguing to note that in our sensitivity analyses (Table S1), mothers with NVP admitted into the hospital and received treatment have children with similar outcomes as those born to mothers with no, mild, or moderate NVP. Consistent with two prior studies, poorer outcomes are identified in children whose mothers have not sought aggressive treatment for NVP.^26^, ^27^ As further described below, lack of treatment for NVP likely entrenches the condition of persistent ketonuria and starvation during pregnancy, while early rehydration or use of antiemetics may help to restore a normal *in utero* environment.

In this study, mothers with severe NVP also have infants born at earlier gestational age. This corroborates previously established risk factors and reinforces the need for awareness regarding the psychosocial burden of NVP.^10^, ^12^, ^13^, ^14^, ^15^, ^16^, ^49^ The exact mechanisms which drive NVP remain an area of investigation. Emerging evidence suggests a multifactorial pathogenesis involving an interaction between genetic, hormonal, and gastrointestinal factors.^50^ Previously, NVP was attributed to hCG production; however, a review of 31 papers showed conflicting evidence.^51^ There has been more support for the role of genetic factors in the pathogenesis of NVP. A genome‐wide association study (GWAS) of European mothers identifies GDF15 (encoding growth/differentiation factor 15), which delays gastric emptying and contributes to nausea, as a risk factor. GFRAL, the receptor of GDF15, in the vomiting centre of the brainstem (ie postrema), which signals loss of appetite and taste aversion, is another risk factor.^52^ The GWAS also implicates IGFBP7 (encoding insulin‐like growth factor‐binding protein 7), a placental protein and biomarker of cachexia, and PGR, a hormone receptor involved in reducing gastrointestinal motility during pregnancy, in the aetiology of NVP. Interestingly, all three identified risk genes (GDF15, IGFBP7, and PGR) are expressed in the placenta, which points to the importance of a placental component of NVP. Additionally, a familial aggregation study has found that NVP risk is increased in women with affected mothers or sisters, again strongly suggesting a genetic association in NVP.^11^ A GWAS study related to this topic in Asia may prove valuable for elucidating the genetic bases of these interethnic differences and may open new opportunities for modulation of NVP.

A possible explanation for the associations between NVP and neurobehavioural outcomes of children relates to the “foetal programming hypothesis,”^22^ which posits that negative stimuli to the foetus during a critical window of development can programme long‐term health. Indeed, severe vomiting in pregnancy is often associated with rapid weight loss, malnutrition, poor diet quality,^22^ restricted eating patterns, and ketonuria (which is a marker of acute starvation), potentially mimicking a state of famine. Studies have suggested that such phenotypic pliability may be enacted through epigenetic modifications, for example DNA methylation in utero.^53^ To paint a more conclusive picture regarding the epigenetic alterations induced by NVP in the offspring, epigenome‐wide association studies should be undertaken to identify differences in genome methylation between children with or without exposure to NVP.

In contrast to the observation that hyperemesis gravidarum is associated with worse placental dysfunction^13^, ^21^ and negatively influences offspring development,^20^, ^23^, ^24^, ^25^ there is growing acceptance of the idea that milder symptoms may be protective of pregnancy outcomes such as miscarriage and congenital malformation.^17^, ^19^, ^54^ These ostensibly opposing effects on perinatal outcomes allude to the complex biology of NVP. Further studies are warranted to understand the possibility of NVP providing feto‐protection while *in utero* and simultaneously setting an unfavourable development trajectory in the offspring after birth.

## 5 CONCLUSIONS

Severe NVP is highly prevalent in this Asian cohort and is associated with a multitude of unfavourable neurobehavioural outcomes in the offspring.

## CONFLICT OF INTEREST

S‐Y Chan, KM Godfrey, and Y‐S Chong are part of the Epigen Academic Consortium that has received funding for research studies outside of this submitted work. All other authors declare no conflicts of interest.

## Supporting information

Supplementary Material

## References

[1] Parker S , Starr J , Collett B , Speltz M , Werler M . Nausea and vomiting during pregnancy and neurodevelopmental outcomes in offspring. Paediatr Perinat Epidemiol. 2014;28:527‐535. 25327160 10.1111/ppe.12151 PMC4232991

[2] Practice Bulletin No. 153: nausea and vomiting of pregnancy. Obstet Gynecol. 2015;126:e12‐e24. 26287788 10.1097/AOG.0000000000001048

[3] O'Brien B , Zhou Q . Variables related to nausea and vomiting during pregnancy. Birth (Berkeley, Calif). 1995;22:93‐100. 10.1111/j.1523-536x.1995.tb00566.x 7779229

[4] Matsuo K , Ushioda N , Nagamatsu M , Kimura T . Hyperemesis gravidarum in Eastern Asian population. Gynecol Obstet Invest. 2007;64:213‐216. 17664884 10.1159/000106493

[5] Vikanes A , Grjibovski A , Vangen S , Magnus P . Variations in prevalence of hyperemesis gravidarum by country of birth: a study of 900,074 pregnancies in Norway, 1967–2005. Scand J Public Health. 2008;36:135‐142. 18519277 10.1177/1403494807085189

[6] Zhang J , Cai W . Severe vomiting during pregnancy: antenatal correlates and fetal outcomes. Epidemiology (Cambridge, Mass). 1991;2:454‐457. 1790200

[7] Goodwin T . Hyperemesis gravidarum. Clin Obstet Gynecol. 1998;41:597‐605. 9742356 10.1097/00003081-199809000-00014

[8] Fairweather DV . Nausea and vomiting in pregnancy. Am J Obstet Gynecol. 1968;102:135‐175. 4877794 10.1016/0002-9378(68)90445-6

[9] Niebyl J . Clinical practice. Nausea and vomiting in pregnancy. N Engl J Med. 2010;363:1544‐1550. 20942670 10.1056/NEJMcp1003896

[10] Verberg M , Gillott D , Al‐Fardan N , Grudzinskas J . Hyperemesis gravidarum, a literature review. Hum Reprod Update. 2005;11:527‐539. 16006438 10.1093/humupd/dmi021

[11] Zhang Y , Cantor RM , MacGibbon K , et al. Familial aggregation of hyperemesis gravidarum. Am J Obstet Gynecol. 2011;204(230):e231‐e237. 10.1016/j.ajog.2010.09.018 PMC3030697 20974461

[12] Attard CL , Kohli MA , Coleman S , et al. The burden of illness of severe nausea and vomiting of pregnancy in the United States. Am J Obstet Gynecol. 2002;186:S220‐S227. 12011890 10.1067/mob.2002.122605

[13] Buyukkayaci ND , Ozcan O , Bostanci M . Hyperemesis gravidarum affects maternal sanity, thyroid hormones and fetal health: a prospective case control study. Arch Gynecol Obstet. 2015;292:307‐312. 25638450 10.1007/s00404-015-3632-2

[14] Davis M . Nausea and vomiting of pregnancy: an evidence‐based review. J Perinat Neonatal Nurs. 2004;18:312‐328. 15646303 10.1097/00005237-200410000-00002

[15] Ezberci İ , Güven E , Ustüner I , Sahin F , Hocaoğlu C . Disability and psychiatric symptoms in hyperemesis gravidarum patients. Arch Gynecol Obstet. 2014;289:55‐60. 23807698 10.1007/s00404-013-2934-5

[16] Mazzotta P , Maltepe C , Navioz Y , Magee L , Koren G . Attitudes, management and consequences of nausea and vomiting of pregnancy in the United States and Canada. Int J Gynaecol Obstet. 2000;70:359‐365. 10967171 10.1016/s0020-7292(00)00255-1

[17] Chortatos A , Haugen M , Iversen PO , et al. Pregnancy complications and birth outcomes among women experiencing nausea only or nausea and vomiting during pregnancy in the Norwegian Mother and Child Cohort Study. BMC Pregnancy Childbirth. 2015;15:138. 26100060 10.1186/s12884-015-0580-6 PMC4477493

[18] Goodwin T . Nausea and vomiting of pregnancy: an obstetric syndrome. Am J Obstet Gynecol. 2002;186:S184‐S189. 12011884 10.1067/mob.2002.122592

[19] Hinkle SN , Mumford SL , Grantz KL , et al. Association of nausea and vomiting during pregnancy with pregnancy loss: a secondary analysis of a randomized clinical trial. JAMA Intern Med. 2016;176:1621‐1627. 27669539 10.1001/jamainternmed.2016.5641 PMC6191846

[20] Nulman I , Rovet J , Barrera M , Knittel‐Keren D , Feldman B , Koren G . Long‐term neurodevelopment of children exposed to maternal nausea and vomiting of pregnancy and diclectin. J Pediatr. 2009;155:45‐50, 50. e41–42. 19394042 10.1016/j.jpeds.2009.02.005

[21] Hu R , Chen Y , Zhang Y , et al. Association between vomiting in the first trimester and preterm birth: a retrospective birth cohort study in Wuhan, China. BMJ Open. 2017;7:e017309. 10.1136/bmjopen-2017-017309 PMC5623485 28963301

[22] Barker DJ . The developmental origins of adult disease. J Am Coll Nutr. 2004;23:588s‐595s. 15640511 10.1080/07315724.2004.10719428

[23] Fejzo M , Magtira A , Schoenberg F , Macgibbon K , Mullin P . Neurodevelopmental delay in children exposed in utero to hyperemesis gravidarum. Eur J Obstet Gynecol Reprod Biol. 2015;189:79‐84. 25898368 10.1016/j.ejogrb.2015.03.028

[24] Martin R , Wisenbaker J , Huttunen M . Nausea during pregnancy: relation to early childhood temperament and behavior problems at twelve years. J Abnorm Child Psychol. 1999;27:323‐329. 10503649 10.1023/a:1022662726587

[25] Mullin PM , Bray A , Schoenberg F , et al. Prenatal exposure to hyperemesis gravidarum linked to increased risk of psychological and behavioral disorders in adulthood. J Dev Origins Health Dis. 2011;2:200‐204. 10.1017/S2040174411000249 25141163

[26] Nulman I , Maltepe C , Farine D , Koren G . Neurodevelopment of children after maternal hospitalization for nausea and vomiting of pregnancy [252]. Obstet Gynecol. 2015;125:81S.

[27] Koren G , Cohen R . Effect of hyperemesis gravidarum on child neurodevelopment. Australas Med J. 2018;11:492‐496.

[28] Getahun D , Fassett MJ , Jacobsen SJ , et al. Autism spectrum disorders in children exposed in utero to Hyperemesis Gravidarum. Am J Perinatol. 2019 10.1055/s-0039-1696670 31581303

[29] Hisle‐Gorman E , Susi A , Stokes T , Gorman G , Erdie‐Lalena C , Nylund C . Prenatal, perinatal, and neonatal risk factors of autism spectrum disorder. Pediatr Res. 2018;84:190‐198. 29538366 10.1038/pr.2018.23

[30] Whitehouse AJO , Alvares GA , Cleary D , et al. Symptom severity in autism spectrum disorder is related to the frequency and severity of nausea and vomiting during pregnancy: a retrospective case‐control study. Mol Autism. 2018;9:37. 29951183 10.1186/s13229-018-0223-7 PMC6009817

[31] Fejzo M , Kam A , Laguna A , MacGibbon K , Mullin P . Analysis of neurodevelopmental delay in children exposed in utero to hyperemesis gravidarum reveals increased reporting of autism spectrum disorder. Reprod Toxicol. 2018;84:59‐64. 30594672 10.1016/j.reprotox.2018.12.009

[32] Soh S‐E , Tint MT , Gluckman PD , et al. Cohort profile: Growing Up in Singapore Towards healthy Outcomes (GUSTO) birth cohort study. Int J Epidemiol. 2014;43:1401‐1409. 23912809 10.1093/ije/dyt125

[33] First MB , Spencer E , Spencer E , et al. Infant‐toddler social and emotional assessment (ITSEA) In: Volmer FR , ed. Encyclopedia of Autism Spectrum Disorders. New York, NY: Springer New York; 2013:1601‐1606.

[34] Allison C , Baron‐Cohen S , Wheelwright S , et al. The Q‐CHAT (Quantitative CHecklist for Autism in Toddlers): a normally distributed quantitative measure of autistic traits at 18–24 months of age: preliminary report. J Autism Dev Disord. 2008;38:1414‐1425. 18240013 10.1007/s10803-007-0509-7

[35] Magiati I , Goh DA , Lim SJ , et al. The psychometric properties of the Quantitative‐Checklist for Autism in Toddlers (Q‐CHAT) as a measure of autistic traits in a community sample of Singaporean infants and toddlers. Mol Autism. 2015;6:40. 26124950 10.1186/s13229-015-0032-1 PMC4484636

[36] Bayley N , Psychological C . Bayley Scales of Infant Development. New York, NY: Psychological Corp; 1993.

[37] Achenbach TM . Child Behavior Checklist. Encyclopedia of Clinical Neuropsychology. New York, NY: Springer New York; 2011:546‐552.

[38] Kaufman AS , Kaufman NLK . Brief Intelligence Test‐Second Edition (KBIT‐2). Bloomington, MN: Pearson Inc; 2004.

[39] Meades R , Ayers S . Anxiety measures validated in perinatal populations: a systematic review. J Affect Disord. 2011;133:1‐15. 21078523 10.1016/j.jad.2010.10.009

[40] Spielberger CD . State‐Trait anxiety inventory In: Weiner IB , Craighead WE , eds. The Corsini encyclopedia of psychology. Hoboken, NJ: John Wiley & Sons, Inc; 2010.

[41] Steer R , Ball R , Ranieri W , Beck A . Dimensions of the Beck Depression Inventory‐II in clinically depressed outpatients. J Clin Psychol. 1999;55:117‐128. 10100838 10.1002/(sici)1097-4679(199901)55:1<117::aid-jclp12>3.0.co;2-a

[42] Steer R , Ball R , Ranieri W , Beck A . Further evidence for the construct validity of the Beck depression Inventory‐II with psychiatric outpatients. Psychol Rep. 1997;80:443‐446. 9129364 10.2466/pr0.1997.80.2.443

[43] Cox J , Holden J , Sagovsky R . Detection of postnatal depression. Development of the 10‐item Edinburgh Postnatal Depression Scale. Br J Psychiatry. 1987;150:782‐786. 3651732 10.1192/bjp.150.6.782

[44] Matthey S , Ross‐Hamid C . Repeat testing on the Edinburgh Depression Scale and the HADS‐A in pregnancy: differentiating between transient and enduring distress. J Affect Disord. 2012;141:213‐221. 22695259 10.1016/j.jad.2012.02.037

[45] Phua DY , Kee MKZL , Koh DXP , et al. Positive maternal mental health during pregnancy associated with specific forms of adaptive development in early childhood: Evidence from a longitudinal study. Dev Psychopathol. 2017;29:1573‐1587. 29162171 10.1017/S0954579417001249

[46] StataCorp LP . Stata, Software: Release 13. College Station, TX: StataCorp LP; 2013.

[47] Von Hippel PT . 4. Regression with missing Ys: an improved strategy for analyzing multiply imputed data. Sociol Methodol. 2007;37:83‐117.

[48] Department Of Statistics, Singapore . General Household Survey 2015. https://www.singstat.govsg/‐/media/files/publications/ghs/ghs2015/findings.pdf. Last accessed 2 Oct 2019. 2015

[49] Hizli D , Kamalak Z , Kosus A , Kosus N , Akkurt G . Hyperemesis gravidarum and depression in pregnancy: is there an association? J Psychosom Obstet Gynaecol. 2012;33:171‐175. 22946891 10.3109/0167482X.2012.717129

[50] Bustos M , Venkataramanan R , Caritis S . Nausea and vomiting of pregnancy‐What's new? Auton Neurosci. 2017;202:62‐72. 27209471 10.1016/j.autneu.2016.05.002 PMC5107351

[51] Niemeijer MN , Grooten IJ , Vos N , et al. Diagnostic markers for hyperemesis gravidarum: a systematic review and metaanalysis. Am J Obstet Gynecol. 2014;211(150):e1‐e150. 10.1016/j.ajog.2014.02.012 24530975

[52] Fejzo MS , Trovik J , Grooten IJ , et al. Nausea and vomiting of pregnancy and hyperemesis gravidarum. Nat Rev Dis Primers. 2019;5:62. 31515515 10.1038/s41572-019-0110-3

[53] Chmurzynska A . Fetal programming: link between early nutrition, DNA methylation, and complex diseases. Nutr Rev. 2010;68:87‐98. 20137054 10.1111/j.1753-4887.2009.00265.x

[54] Koren G , Madjunkova S , Maltepe C . The protective effects of nausea and vomiting of pregnancy against adverse fetal outcome—a systematic review. Reprod Toxicol. 2014;47:77‐80. 24893173 10.1016/j.reprotox.2014.05.012

