# Supplementary material for: Severity of nausea and vomiting in pregnancy and early childhood neurobehavioural outcomes: The Growing Up in Singapore Towards Healthy Outcomes study
Source: Paediatr Perinat Epidemiol. 2020 Jun 23;35(1):98–108. doi: 10.1111/ppe.12703 (PMC7116637; doi:10.1111/ppe.12703)
Supplement: Supplementary file 1 — Supplementary Material [file PPE-35-98-s001.docx]

**eTable 1: In the sensitivity analyses, severe NVP without hospital admission is associated with a wider range of child outcomes than severe NVP with hospital admission**

| **Outcome** | **Severity of vomiting** | **Model-adjusted means (95%CI)** | **Adjusted difference in means (95% CI)^a^** |
| --- | --- | --- | --- |
| 1-year ITSEA Activity & impulsivity | None | 5.08 (4.85, 5.30) | 0.0 (Reference) |
|  | Severe: with admission | 6.00 (5.31, 6.69) | 0.9 (-0.2, 1.7) |
|  | Severe: no admission | 6.28 (5.24, 7.32) | 1.2. (0.1, 2.3) |
| 1-year ITSEA Externalizing Behaviours | None | 10.05 (9.48, 10.61) | 0.0 (Reference) |
|  | Severe: with admission | 11.48 (8.85, 14.11 ) | 1.5 (-1.3, 4.3) |
|  | Severe: no admission | 12.77 (11.05, 14.49) | 2.7 (0.9, 4.5) |
| 1-year ITSEA Negative emotionality | None | 7.16 (6.83, 7.49) | 0.0 (Reference) |
|  | Severe: with admission | 8.95 (7.70, 10.20) | 1.8 (0.5, 3.1) |
|  | Severe: no admission | 9.67 (7.85, 11.48) | 2.0 (0.1, 4.0) |
| 1-year ITSEA Sleep problems | None | 2.44 (2.26, 2.61) | 0.0 (Reference) |
|  | Severe: with admission | 2.96 (2.10, 3.82) | 0.5 (-0.5, 1.4) |
|  | Severe: no admission | 3.18 (2.59, 3.77) | 0.7 (0.1, 1.4) |
| 1-year ITSEA Dysregulation | None | 16.57 (15.88, 17.26) | 0.0 (Reference) |
|  | Severe: with admission | 21.38 (17.72, 25.04) | 4.2 (0.3, 8.2) |
|  | Severe: no admission | 20.40 (17.92, 22.88) | 3.8 (1.2, 6.4) |
| 1.5-year QCHAT total | None | 36.09 (35.89, 36.29) | 0.0 (Reference) |
|  | Severe: with admission | 38.28 (37.03, 39.49) | 2.2 (1.2, 3.3) |
|  | Severe: no admission | 39.57 (38.30, 40.84) | 3.5 (2.2, 4.8) |
| 2-year CBCL Emotional reactivity | None | 2.30 (2.22, 2.38) | 0.0 (Reference) |
|  | Severe: with admission | 3.30 (3.07, 3.53) | 1.1 (0.8, 1.4) |
|  | Severe: no admission | 4.10 (3.79, 4.42) | 1.6 (1.2, 2.0) |
| 2-year CBCL Attention problems | None | 2.98 (2.92, 3.04) | 0.0 (Reference) |
|  | Severe: with admission | 3.06 (2.88, 3.24) | 0.3 (-0.1, 0.6) |
|  | Severe: no admission | 3.76 (3.48, 4.04) | 0.8 (0.5, 1.1) |
| 2-year CBCL Sleep problems | None | 3.08 (2.30, 3.16) | 0.0 (Reference) |
|  | Severe: with admission | 3.00 (2.75, 3.24) | 0.3 (-0.1, 0.6) |
|  | Severe: no admission | 4.63 (4.25, 5.01) | 1.6 (1.2, 1.9) |
| 2-year CBCL DSM ADHD | None | 4.91(4.83 ,4.99) | 0.0 (Reference) |
|  | Severe: with admission | 5.50 (5.27, 5.73) | 1.2 (-0.2, 2.0) |
|  | Severe: no admission | 7.01 (6.65, 7.37) | 2.1 (1.7, 2.5) |
| 2-year CBCL DSM affective | None | 2.56 (2.47, 2.65) | 0.0 (Reference) |
|  | Severe: with admission | 2.07( 1.82, 2.33) | -0.3 (-0.6, 0.1) |
|  | Severe: no admission | 5.25 (4.85, 5.64) | 2.7 (2.3, 3.1) |
| 4-year CBCL anxious-depressive (paternal report) | None | 2.90 (2.83, 2.96) | 0.0 (Reference) |
|  | Severe: with admission | 2.96 (2.75, 3.17) | -0.2 (-0.4, 0.1) |
|  | Severe: no admission | 3.75 (3.53, 3.96) | 0.9 (0.6, 1.1) |
| 4-year CBCL anxious-depressive (maternal report) | None | 3.07 (3.01, 3.12) | 0.0 (Reference) |
|  | Severe: with admission | 2.85 (2.66, 3.03) | -0.4 (-0.7, 0.2) |
|  | Severe: no admission | 4.15 (3.96, 4.33) | 1.1 (0.9, 1.3) |
| 4-year CBCL DSM Affective (maternal report) | None | 2.53 (2.47, 2.59) | 0.0 (Reference) |
|  | Severe: with admission | 2.82 (2.62, 3.01) | 0.1 (-0.1, 0.3) |
|  | Severe: no admission | 3.26 (3.07, 3.46) | 0.7 (0.5, 0.9) |
| 4-year CBCL DSM Anxiety (maternal report) | None | 3.82 (3.76 ,3.88) | 0.0 (Reference) |
|  | Severe: with admission | 3.59 (3.39, 3.78) | -0.5 (-0.8, 0.2) |
|  | Severe: no admission | 5.22 (5.03, 5.41) | 1.4 (1.2, 1.6) |
| ^a^ Covariates included gestational age, birthweight Z-scores, maternal age, household monthly income, gestational diabetes, hypertensive disorders of pregnancy, history of smoking exposure and/or positive plasma cotinine, sex of child, maternal general mood factor from pregnancy to 3-month post-partum, and parity.  Abbreviations: ITSEA, Infant Toddler Social and Emotional Assessment; Q-CHAT, Quantitative Checklist for Autism in Toddlers; CBCL, Child Behavior Checklist; DSM, Diagnostic and Statistical Manual of Mental Disorders | | | |

**eTable 2: Comparison between participants with complete data and missing data based on demographic data and early medical variables**

| **Characteristic** | | **1-year complete**  **(N=542)** | **1-year missing (N=630)** | **2-year complete (N=397)** | **2-year missing (N=775)** | **4.5-year complete (N=476)** | **4.5-year missing (N=696)** |
| --- | --- | --- | --- | --- | --- | --- | --- |
| **Severity of nausea and vomiting (NVP)** | **No** | 25.2 | 25.3 | 23.7 | 26.1 | 24.2 | 26.0 |
|  | **Mild-moderate** | 59.4 | 57.8 | 62.3 | 56.6 | 62.0 | 56.1 |
|  | **Severe** | 15.4 | 16.9 | 14.0 | 17.3 | 13.8 | 17.9 |
| **Ethnicity** | **Chinese** | 64.4 | 51.7 | 56.8 | 56.2 | 56.0 | 56.5 |
|  | **Malay** | 21.7 | 27.3 | 27.5 | 24.4 | 27.9 | 24.0 |
|  | **Indian** | 13.9 | 21.0 | 15.7 | 19.4 | 16.1 | 19.5 |
| **Male sex** | | 50.9 | 54.1 | 51.6 | 53.2 | 52.3 | 52.9 |
| **Gestational diabetes during pregnancy** | | 18.1 | 20.1 | 19.5 | 19.1 | 18.1 | 19.9 |
| **Pregnancy-induced hypertension** | | 5.3 | 6.5 | 4.8 | 6.4 | 5.7 | 6.0 |
| **Smoke exposure (self-report and/or positive plasma cotinine level)** | | 14.9 | 14.6 | 17.2 | 13.6 | 18.5 | 12.4 |
| **Number of siblings** | **0** | 45.5 | 45.2 | 45.3 | 45.3 | 46.4 | 44.6 |
|  | **1** | 33.7 | 35.3 | 30.5 | 36.6 | 29.0 | 38.2 |
|  | **2 or more** | 21.8 | 19.5 | 24.2 | 18.1 | 24.6 | 17.2 |
| **Gestational age in weeks, mean (SD)** | | 38.83 (1.45) | 38.41 (2.07) | 38.86 (1.29) | 38.46 (2.03) | 38.83 (1.33) | 38.44 (2.08) |
| **Birthweight Z-score, mean (SD)** | | 0.16 (1.20) | 0.10 (1.26) | 0.12 (1.20) | 0.13 (1.24) | 0.16 (1.21) | 0.10 (1.24) |
